# A novel ZnO@Ag@Polypyrrole hybrid composite evaluated as anode material for zinc-based secondary cell

**DOI:** 10.1038/srep24471

**Published:** 2016-04-14

**Authors:** Jianhang Huang, Zhanhong Yang, Zhaobin Feng, Xiaoe Xie, Xing Wen

**Affiliations:** 1 School of Material Science and Engineering, Nanchang Hangkong University, Nanchang 330063, China; 2 College of Chemistry and Chemical Engineering, Central South University, Changsha 410083, China; 3 Innovation base of energy and chemical materials for graduate students training, Central South University, Changsha 410083, China

## Abstract

A novel ZnO@Ag@Polypyrrole nano-hybrid composite has been synthesized with a one-step approach, in which silver-ammonia complex ion serves as oxidant to polymerize the pyrrole monomer. X-ray diffraction (XRD) and infrared spectroscopy (IR) show the existence of metallic silver and polypyrrole. The structure of nano-hybrid composites are characterized by scanning electron microscope (SEM) and transmission electron microscope (TEM), which demonstrates that the surface of ZnO is decorated with nano silver grain coated with polypyrrole. When evaluated as anode material, the silver grain and polypyrrole layer not only suppress the dissolution of discharge product, but also helps to uniform electrodeposition due to substrate effect and its good conductivity, thus shows better cycling performance than bare ZnO electrode does.

The increasing needs for power sources and the rapid consumption of fossil fuels make the battery systems, which are considered as an important storage of intermittent renewable, play a growing more important role in our daily life. Although there are many different battery systems in market, some problems, such as the low energy densities and environment problem for lead-acid battery, the high cost and flammable nature for Li ion battery^1^,^2^,^3^, make them unqualified for different applications. On the other hand, aqueous battery system is safe and easy to assemble. Among traditional primary battery, zinc is the most widely used anode material for various kinds of primary systems for its unique attributes such as low equivalent weight, good reversibility, high specific energy density, abundant resource and low toxicity. Moreover, it has a large over-potential for hydrogen evolution reaction, which indicates that it possesses good stability in aqueous or alkaline electrolyte with no significant corrosion^4^,^5^,^6^,^7^,^8^.

But during the charge/discharge process, the high dissolution of discharge product of zinc electrode in alkaline electrolyte and its non-uniform deposition will lead to the change of electrode shape and dendrite growth, which can puncture separator and leads to an internal short circuit of battery system in the worst situation^9^,^10^,^11^. A lot of approaches have been tried to slow down or suppress dendrite growth, for instance, introducing additives such as metal oxides^12^,^13^,^14^,^15^,^16^,^17^, surfactants^18^,^19^ and polymers^20^,^21^ into electrode or electrolyte could effectively affect the electrochemical properties of zinc electrode, suppress the shape change and the growth of dendrite. Wang *et al.*^22^ studied the effectiveness of bismuth ion and tetrabutylammonium bromide (TBAB) in inhibiting the formation of zinc dendrite in alkaline electrolyte. They found that Bi_3_^+^ can inhibit the dendritic growth by substrate effect, furthermore, obvious synergistic effect of Bi_3_^+^ and TBAB is another important factor to suppress the formation of dendrite. Calcium hydroxide has long been considered as an effective additive, it can react with zincate ions to form insoluble compound, thereby maintaining zinc species around the electrode^23^. Furthermore, calcium zincate itself can also be used as electrode active material, it presents an improvement of electrochemical properties compared to those of conventional ZnO^24^,^25^. In addition, surface decorating of zinc metal or zinc oxide with other materials is regarded as a more effective strategy to improve comprehensive properties of zinc electrode. Lee *et al.*^26^ synthesized TiO_2_-coated ZnO through a sol-gel method, the TiO_2_ upon the surface of ZnO forms a passive layer, which keeps the electrode away from dissolving into electrolyte. Other than metal oxides mentioned above, organic materials are considered as a kind of effective material to retain the zinc species around vicinity of zinc electrode. Vatsalarani *et al.*^27^,^28^ found that polyaniline coating on zinc electrode could allow the movement of hydroxide ions, but at the same time, it restricted the diffusion of zincate ions due to its fine porous structure and the re-complexation between polymer and zincate ions. Yang *et al.*^29^ prepared ZnO-Polypyrrole complex with assistance of ultrasound during polymerization of pyrrole, and the dendrite growth was effectively suppressed.

In this study, we prepare ZnO@Ag@PPy nano-hybrid composite for the purpose of improving cycle performance of zinc electrode. The effects of surface decoration on the cycling performances of zinc electrode were investigated. As we know that the nano-silver particles possess excellent electron conductivity, furthermore, the polypyrrole could decrease the solubility of discharge product through the re-complexation between zinc species and imine functional group of polypyrrole, so leading to the mitigation of dendritic growth, which make it reasonable that the nano-hybrid composite can exhibit improved cycling performance than bare ZnO.

## Experimental Section

### Material preparation

Zinc oxide (ZnO, ≥99%, AR) was obtained from Xilong chemical Co., Ltd, Ammonia solution (NH_3_, 25–28%, AR) was purchased from Chongqing Chuandong Chemical (Group) Co., Ltd, Silver nitrate (AgNO_3_, ≥99.8%, AR) and Pyrrole (≥99%. CP) was obtained from Sinopharm Chemical Reagent Co., Ltd. Pyrrole suffered vacuum distillation before use. Silver-ammonia solution was prepared with diluted ammonia solution (about 2%) and was used immediately after its preparation. In this paper, the ZnO@Ag@PPy nano-hybrid composites were prepared by using the oxidation-reduction reactions which takes place between silver-ammonia ion and pyrrole. The typical preparation process was described as follow: 600 mg ZnO was dispersed in 20 mL distilled water under vigorous stirring and ultrasound, a fresh silver-ammonia solution prepared with 50, 100, 150 mg AgNO_3_ respectively (the according end product was coded as sample 1, sample 2, sample 3 respectively) was added quickly into above ZnO suspension liquid, afterwards, enough pyrrole monomer was introduced to above system. After another 5 mins ultrasonic treatment, the mixture solution was treated with continuous stirring for 10 h to complete redox reaction. During the reaction process, it was obvious that the color of the system changed from white to brownish black gradually. Finally, we filtrated the resulting suspension to collect the final product, and washed thoroughly using distilled water and methanol, then dried the product in a vacuum oven under 333 K for further characterization and use.

### Characterization of ZnO@Ag@PPy hybrid composite

FT-IR of ZnO@Ag@PPy hybrid composite was performed on Nicolet Nexus-670 FT-IR spectroscope with KBr pellets. SEM (JSM-6360LV) and TEM (JEOL-2010) were employed to record the morphology and structure of test samples. XRD spectrum was recorded on a Philips X’ Pert Pro diffractometer, and Cu Kα served as radiation source to characterize the crystal structures of all the samples.

### Preparation of testing electrodes

For fabrication of zinc electrode, 85 wt. % of ZnO powder was mixed with 10 wt. % acetylene black (served as conductive agent) and 5 wt. % PTFE (served as binder), respectively. And the mixture was grinded with agate mortar until it became muddy mixture with proper viscosity. Then the above muddy mixture was incorporated to a copper mesh used as current collector. For the fabrication of hybrid composite electrode, 95 wt. % as-prepared composite was mixed with 5wt. % PTFE, in which acetylene black is abandoned since the existence of silver in the composite could play the conductive agent instead of acetylene black. The obtained electrodes were dried at 333 K in a vacuum oven for further testing.

### Electrodes testing

For galvanostatic charge/discharge cycle test, a two-electrode cell was employed, which includes working electrode and counter electrode, but without reference electrode compared with a three-electrode system. The capacity of chosen counter electrode (sintered Ni(OH)_2_ electrode) was three times higher than working electrode in the aim of making sure that the capacities of cells was controlled by working electrode. In addition, 6.0 M KOH solution with saturated ZnO (0.45 M ZnO + 6.0 M KOH) was employed to be electrolyte, multilayer polypropylene micro-porous membranes used as separator. And the two-electrode system were charged at 1 C (or 3 C) for 60 min (or 20 min) and discharged at 1C (or 3C) down to the cut-off voltage of 1.2 V, where 1C is 4.5 mA cm^−2^ and 3C is 13.5 mA cm^−2^. Cycle test was performed with a battery test apparatus NEWWARE BTS-610 (Newware Technology Co., Ltd., China). For cyclic voltammetry test (CV) and electrochemical impedance spectroscope (EIS), we employ the three-electrode system to investigate the electrochemical performance, in which Hg/HgO electrode served as reference electrode, a sintered Ni(OH)_2_ electrode was used as counter electrode, and as-prepared hybrid composite electrodes served as the working electrode. The CV test was performed over the range from −1.65 V to −0.85 V, at a scanning rate of 20 mV s^−1^. The amplitude for EIS test was set as 5 mV. And EIS result was fitted by the software of Zview. The above testing were conducted with RST 5000 (Zhengzhou Shiruisi Technologh Co., Ltd.) electrochemical workstation.

## Results and Discussion

### Characterization of as-prepared ZnO@Ag@PPy nano-hybrid composite

**Figure 1:**
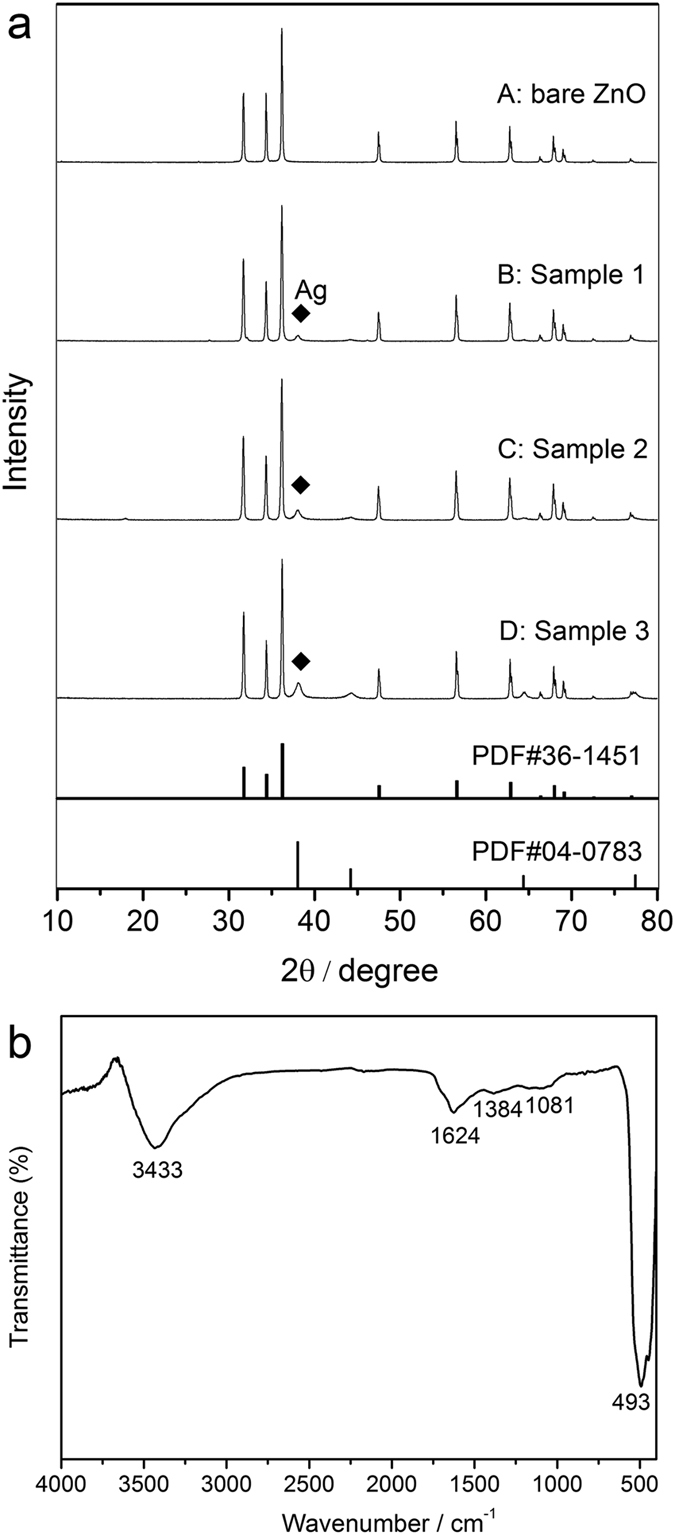
(**a**) XRD pattern for bare ZnO, Sample 1, Sample 2 and Sample 3; (**b**) FT-IR spectrum for sample 2.

The most common method to prepare polypyrrole in a chemical route is to oxidize pyrrole monomer directly with oxidizing agent, such as ferric chloride and ammonium persulfate^30^,^31^. In the redox process, the oxidizing agent, Fe(III), will be reduced to Fe(II), meanwhile, pyrrole monomer will be oxidized to polypyrrole. It is important to note that the standard reduction potential of Fe(III) to Fe(II) is 0.771 V, which gives us a reference to judge whether or not pyrrole can be oxidized by oxidizing agent. In theory, an oxidizing agent has ability to oxidize pyrrole monomer if it possesses higher reduction potential than that of Fe(III)/Fe(II). The standard reduction potential of Ag(I) to Ag(0) is 0.7996 V, which is higher than 0.771 V, so it has the capacity to polymerize pyrrole to polypyrrole, simultaneously, Ag(I) itself will reduce to metallic silver. The XRD pattern of bare ZnO, sample 1, sample 2 and sample 3 are shown in Fig. 1a coded as curve A, B, C, D. From curve A we know that the position of diffraction peaks of bare ZnO match well with that of hexagonal wurtzite ZnO which is shown at the bottom of Fig. 1a (JCPDS card 36–1451). As to ZnO@Ag@PPy nano-hybrid composite, other diffraction peaks corresponding to metal silver observed clearly at 2θ of 38.0°, 44.3°, 64.5° and 77.3° can be observed besides the diffraction peak of bare ZnO (the PDF card 04–0783 for metallic silver is given for comparison), which proved that the silver-ammonia complex ion has been successfully reduced to metallic silver. The retention of ZnO diffraction peaks indicates that the hexagonal wurtzite structure of ZnO in as-prepared nano-hybrid composite has not been destroyed during the preparation process. In addition, it is obvious that the intensity of diffraction peak at 38.0° for nano-hybrid composites increases gradually with the increase of quantity of silver-ammonia solution, this phenomenon reflects the fact that the content of loaded silver in nano-hybrid composite can be controlled by the quantity of original silver-ammonia solution during preparation process. Figure 1b shows the FT-IR spectrum of sample 2. As can be seen there are five remarkable absorption peaks arising at 3433, 1624, 1384, 1081 and 493 cm^−1^, respectively. The strong infrared band arising at 3433 cm^−1^ could be ascribed to the overlap of N–H stretching of polypyrrole and O–H stretching of absorbed water. Furthermore, there is another strong absorption band at 493 cm^−1^, it can be ascribed to the vibration of Zn-O bond because of the high content of ZnO in the nano-hybrid composite. The remaining three absorption peaks at 1624, 1384 and 1081 cm^−1^ can be considered as the characteristic absorption of polypyrrole which corresponds to the vibration of conjungated double bond in pyrrole ring, albeit they shift to higher wavenumber to a certain extent compared with pure polypyrrole reported in previous works. In detail, the absorption peak at 1624 cm^−1^ demonstrates the existence of C=C double bond in pyrrole ring. In addition, the absorption peaks at 1384 and 1082 cm^−1^ can be considered as the C–H in-plane vibration and bending respectively. Above absorption peaks prove the presence of polypyrrole in nano-hybrid composite. The shift of peak location can be ascribed to the interaction between polypyrrole and Ag nano-particles which influences the skeletal vibrations of pyrrole, this phenomenon can also be observed in other polypyrrole/metal oxide composites^32^,^33^.

**Figure 2:**
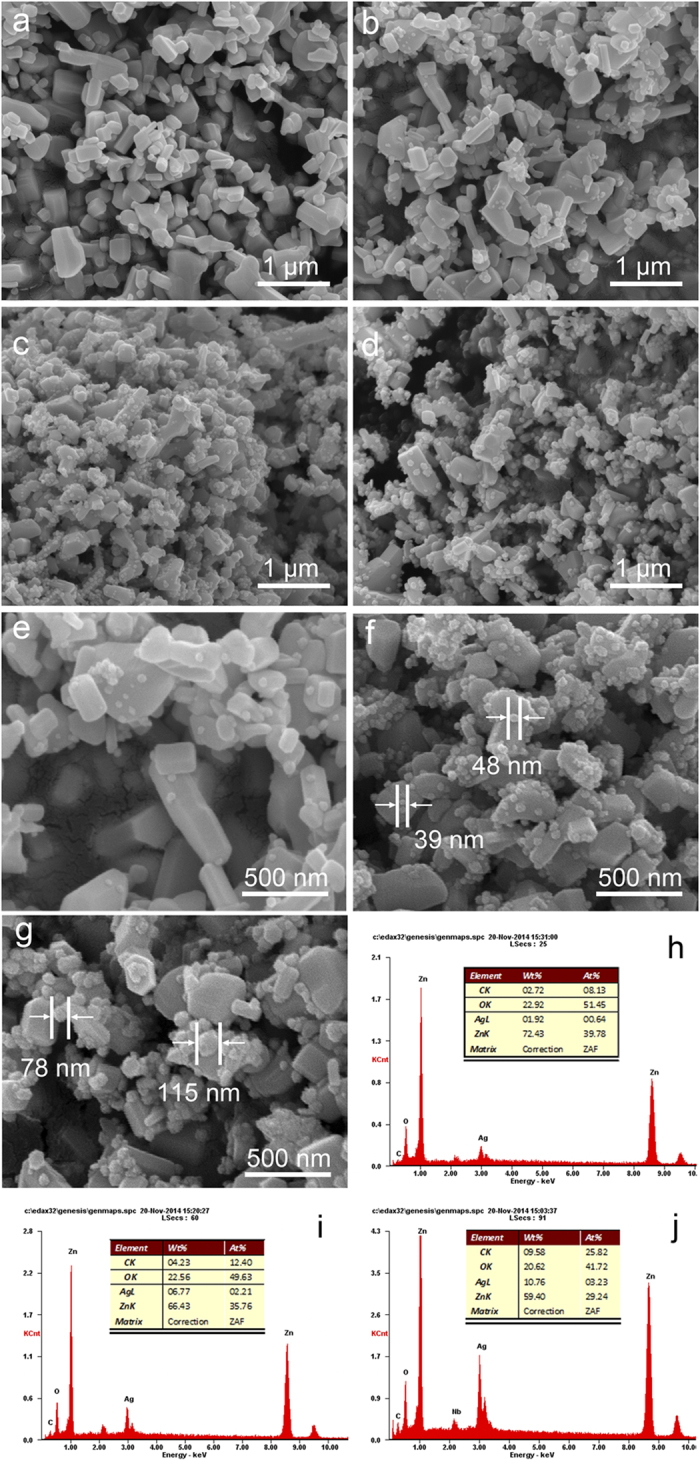
SEM images of (**a**) bare ZnO (**b**) Sample 1 (**c**) Sample 2 and (**d**) Sample 3; Magnified SEM images of (**e**) sample 1, (**f**) Sample 2 and (**g**) Sample 3; EDX analysis for (**h**) Sample 1, (**i**) Sample 2 and (**j**) Sample 3.

**Figure 3:**
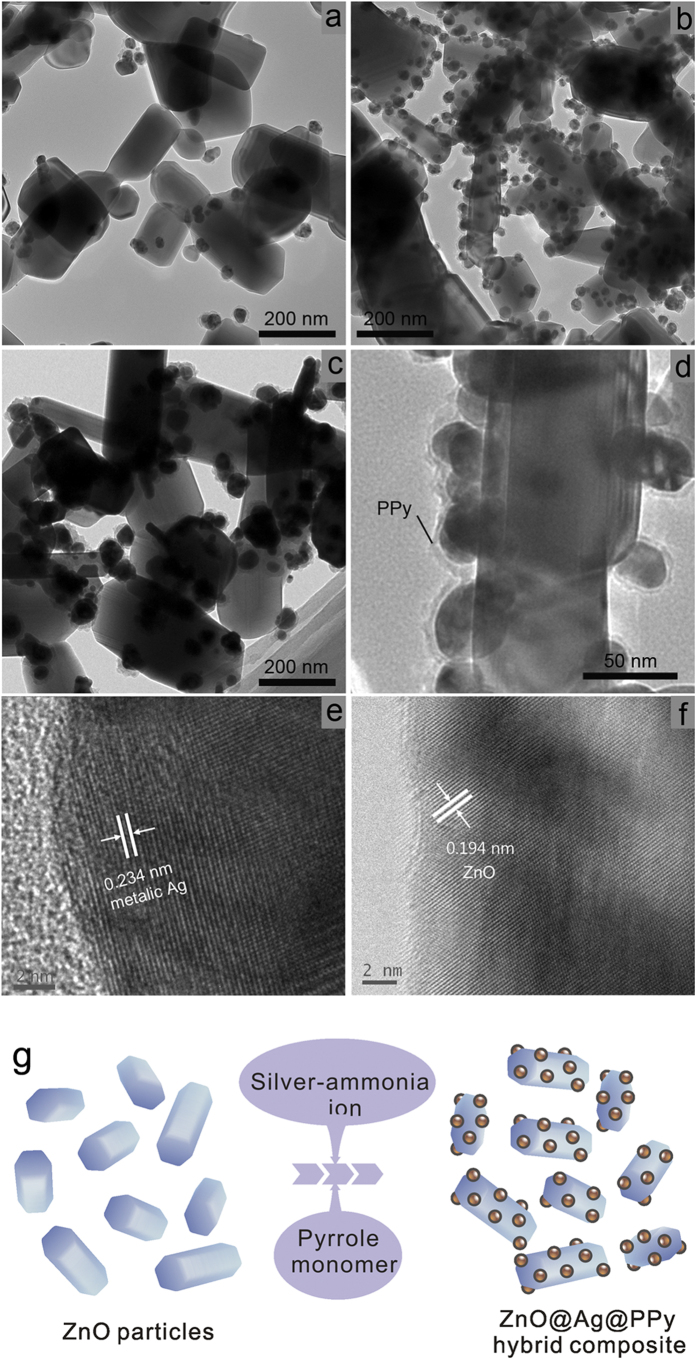
TEM images of (**a**) Sample 1, (**b**) Sample 2 and (**c**) Sample 3; (**d**) Magnified TEM images of Sample 2; (**e**) HR-TEM image of metallic Ag particle in Sample 2; (**f**) HR-TEM image of ZnO particle in Sample 2; (**g**) Schematic diagram of ZnO@Ag@PPy hybrid composite.

Figure 2 shows the SEM images of bare ZnO and ZnO@Ag@PPy nano-hybrid composites. Figure 2a is the bare ZnO with a hexagonal prism morphology whose surface is smooth. Figure 2b,c,d show the SEM image of sample 1, 2, 3 respectively. In Figure 2b, it can be seen that metallic silver is dotted sporadically upon the surface of ZnO particles. The magnified SEM image of sample 1 can be found in Fig. 2e, it provides us a clear vision that there is some tiny metallic silver grain attached on the surface of prism ZnO particles. Figure 2c shows the morphology of sample 2, it is obvious that the quantity of silver grain become larger compared with sample 1, this is because the original addition of silver-ammonia solution for sample 2 is more than that of sample 1. Furthermore, it can be noticed that the size of silver grain is almost unchanged in the comparison between the magnified SEM image of sample 1 (Fig. 2e) and sample 2 (Fig. 2f). But with further increase of original addition of silver-ammonia solution, as sample 3 shown in Fig. 2d, the size of silver grain become larger, which can even reach 115 nm (as shown in Fig. 2g), it was much larger than that of sample 1 and sample 2 (lower than 50 nm). This could be explained as follow: the high concentration of silver-ammonia ion promotes the growth of silver grain rather than the formation of silver nucleus, on the other hand, agglomeration phenomenon of silver grain emerges when the concentration of silver-ammonia ion becomes too high. The EDX analysis for sample 1, 2, 3 are shown in Fig. 3h, i, j, respectively, which reveals the content of metallic silver in the nano-hybrid composites. It is 1.92, 6.77, 10.76% for sample 1, 2, 3, respectively.

TEM and HRTEM analyses have been performed in order to further investigate the structure and component of ZnO@Ag@PPy nano-hybrid composite, and the results are shown in Fig. 3. In details, Fig. 3 a, b, c show the structure of sample 1, sample 2 and sample 3 respectively. The observation is consistent with that recorded by SEM, the surface of ZnO particles is coated with small nano metallic silver grain, and their number become larger with the increasing silver content. Further magnified TEM image shown in Fig. 3d reveals that there is a layer of polypyrrole upon the surface of silver grain, indicating the ZnO@Ag@PPy hybrid composite is synthesized successfully. The HRTEM images of Ag and ZnO particle are shown in Fig. 3e, f, respectively, in which the lattice fringes can be clearly observed. It indicates the crystalline nature of nano-silver particles and ZnO particles. As to metallic silver grain, the distance between the adjacent lattice planes is around 0.234 nm, corresponding to the (111) planes of metallic silver (d_111_ = 0.236 nm). As to ZnO, it is around 0.194 nm, corresponding to (102) planes of hexagonal ZnO (d_102_ = 0.191 nm). In order to iconically understand the key concepts of the material, an illustration of construct of the hybrid composite is provided in Fig. 3g.

### Cyclic voltammetry analysis

**Figure 4:**
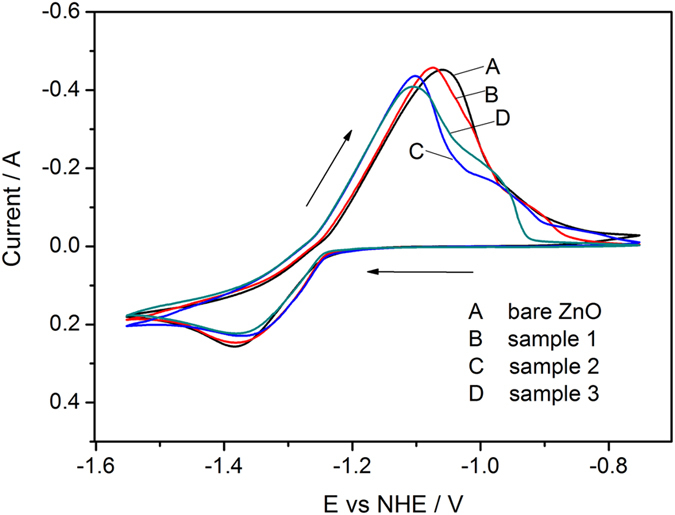
Cyclic voltammograms of bare ZnO (curve A), Sample 1 (curve B), Sample 2 (curve C) and Sample 3 (curve D) at 10th cycle.

Cyclic voltammetry is conducted in order to study electrochemical performance of composite. The testing electrodes were immersed in electrolyte for 6 h to reach equilibration. And the cyclic voltammograms of bare ZnO electrode as well as ZnO@Ag@PPy nano-hybrid composite electrodes at 10th cycle are presented in Fig. 4. During the anodic scan, it is worth noting that the location of anodic peak of sample 2 and sample 3 present a shift towards a more negative potential compared with that of bare ZnO and sample 1. As we know, the peak separation (Δ*E*_a,c_) calculated from the interval between anodic peak potential (*E*_p,a_) and cathodic peak potential (*E*_p,c_) is usually considered as a measurement of reversibility of electrode reaction. Generally, the closer of the potential interval, the better reversibility it has. From Fig. 4, the Δ*E*_a,c_ can be calculated as 0.325, 0.309, 0.271, 0.275 V for bare ZnO, sample 1, sample 2 and sample 3, respectively. The value of Δ*E*_a,c_ for sample 2 and sample 3 is smaller than that for bare ZnO and sample 1, indicating the reversibility of sample 2 and sample 3 is superior to that of bare ZnO and sample 1. The main reason can be ascribed to the decoration upon the surface of ZnO particles, the loaded metallic silver grain and the conductive polymer improve the conductivity of ZnO, make the redox reaction easy to happen.

In addition, another anodic peak appears at a more positive potential for hybrid composites can be observed in anode process, and it becomes more apparent with the increase of loaded Ag. The phenomenon of “two anodic peaks” is a characteristic for zinc electrode reaction, which can be explained by the two processes of anodic dissolution^23^,^34^,^35^. Generally, there are two kinds of anodic electrochemical reactions taking place during the discharge process, which can be described as the following equations:


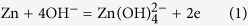



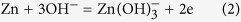


At the beginning of discharge process, the supply of hydroxide ion is abundant, and the electrochemical reaction performances according to equation (1), resulting in the emergence of first anodic peak. With the proceeding of discharge process, the concentration of hydroxide ion at the vicinity of surface of electrode becomes insufficient to maintain the reaction process according to equation (1), on this occasion, equation (2) will perform. As to nano-hybrid composites, the existence of silver and polypyrrole leads to an inadequate contact between active material and hydroxide ion at reaction layer, thus, the lack of hydroxide ion is more serious compared to bare ZnO, and the second anode peak is more obvious than that of bare ZnO.

As to cathodic process, the peak potential of sample 2 and sample 3 present slight shift towards to positive direction compared to bare ZnO and sample 1, which indicates that the hybrid composite has high reduction reaction kinetics because of the existence of Ag nano-particles.

### Cycle performance

**Figure 5:**
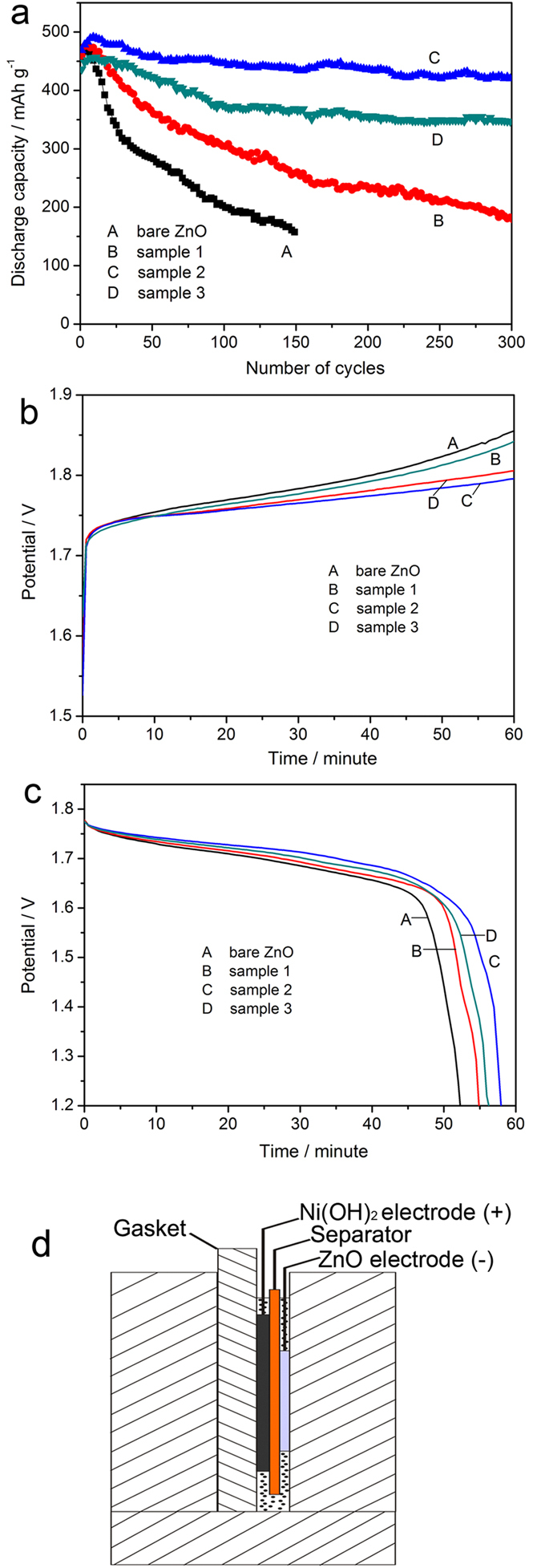
(**a**) Cycle performance of bare ZnO (curve A), Sample 1 (curve B), Sample 2 (curve C) and Sample 3 (curve D) at current rate of 1C; (**b**) charge curves and (**c**) discharge curves of bare ZnO (curve A), Sample 1 (curve B), Sample 2 (curve C) and Sample 3 (curve D) at 10th cycle; (**d**) Scheme of electrochemical cell.

The cycle performance of nano-hybrid composite was investigated by the galvanostatic discharge-charge test at 1 C rate (4.5 mA cm^−2^), and the active mass of electrode is 10 mg. Figure 5a shows the changes of discharge capacity along with the increase of cycle number. As can be seen all the testing electrodes suffer relative lower discharge capacity in initial few cycles. This is due to the fact that the as-prepared electrodes need some cycles to be activated. And after achieving the highest discharge capacity, bare ZnO (curve A) shows a swift drop in the discharge capacity during the first fifty cycles, while the hybrid composite electrodes mitigate the swift drop in different degree, especially sample 2 and sample 3, and they present a much better capacity retention in subsequent cycles. As to bare ZnO electrode, the capacity retention is measured to be as 32.14% after 150 cycles, and there is 151.88 mAh g^−1^ of discharge capacity left. As to hybrid composite electrode, the capacity retention after 150 cycles is 55.09%, 88.79%, 79.91% for sample 1, sample 2, sample 3 respectively, corresponding to the discharge capacity of 264.67, 436.54, 365.19 mAh g^−1^. What is more, after 300 cycles, there is 180.90, 422.98, 345.82 mAh g^−1^ left for sample 1, sample 2, sample 3 respectively. These results clearly demonstrate that the hybrid composite electrodes can effectively alleviate the capacity drop and keep the discharge capacity relative stable along with increase of cycle number. The improvement of hybrid composite in cycle stability is associated with the loaded metallic silver and polypyrrole. First, the metallic silver nanoparticles and polypyrrole on the surface of ZnO reduced the contact of ZnO particles with electrolyte, suppressed the dissolution of ZnO. Second, polypyrrole served as an trapping layer, it better retained the discharge product in the electrode rather than dissolving into electrolyte, because polypyrrole can limit the diffusion of the zinc species due to its re-complexation with zincate ions^27^,^28^. Finally, a substrate effect of metallic silver and polypyrrole is also an important factor. They are insoluble in alkaline electrolyte, and serve as substrate, which influence the zinc electro-deposition during the charge process, suppressing the growth of zinc dendrite. Figure 5b,c show the typical charge and discharge curves of bare ZnO electrode (cuve A), sample 1 (curve B), sample 2 (curve C) and sample 3 (curve D) at tenth cycle. It is obvious that the hybrid composite electrodes show relative lower charge platform as well as higher discharge platform in comparison of bare ZnO electrode, which indicates that the reversibility of hybrid composite electrode is better than that of bare ZnO electrode. The improved reversibility of hybrid composite could be ascribed to the metallic silver nano-particles and polypyrrole upon the surface of zinc oxide particles, whose good conductivity provides good internal electrical contact of electrode, facilitates electron transfer in electrode. It can be noticed that sample 2 shows the best cycle performance. Sample 1 has no sufficient decoration, so the improvement in electrochemical performance is limited. As to sample 3, the excess content of decoration in hybrid composite leads to the decrease of discharge capacity. Furthermore, too much silver and polypyrrole influences the diffusion of hydroxide ion during the electrochemical reaction. This is consistent with the phenomenon of “two anodic peaks” in cyclic voltammetry. So the proper content of decoration is a significant factor to influence electrochemical performance of electrode.

**Figure 6:**
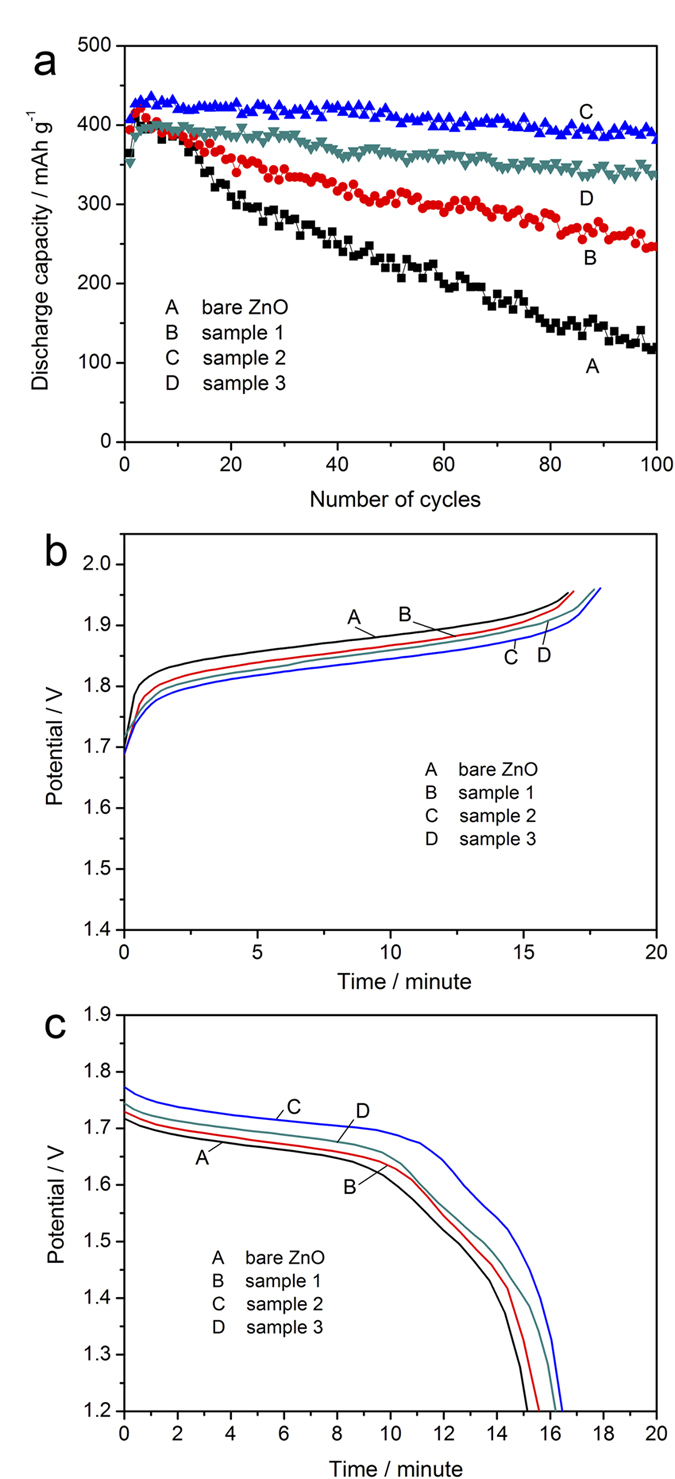
(**a**) Cycle performance of bare ZnO (curve A), Sample 1 (curve B), Sample 2 (curve C) and Sample 3 (curve D) at current rate of 3C; (**b**) charge curves and (**c**) discharge curves of bare ZnO (curve A), Sample 1 (curve B), Sample 2 (curve C) and Sample 3 (curve D) at 10th cycle.

High rate performance is an important rule to evaluate the electrochemical performance for zinc electrode, so the galvanostatic discharge-charge test at 3C rate (13.5 mA cm^−2^) is performed. Figure 6a shows the changes of discharge capacity along with the increase of cycle number. It can be observed that hybrid composite electrodes also presents a more stable cycle performance at high current rate compared to the bare ZnO electrode. Due to the excellent conductivity, the large polarization caused by high current density can be effectively relived in hybrid electrodes. Furthermore, the substrate effect of decoration improves the distribution of electrodeposition of zinc species, which inhibits the growth of zinc dendrite. These are the main reasons for the enhanced performance. The improvement can be also seen in the charge-discharge curves shown in Fig. 6b, c (bare ZnO: curve A, sample 1: curve B, sample 2: curve C, sample 3: curve D). The higher discharge plateau and lower charge platform imply the smaller polarization compared to bare ZnO electrode, which means that ZnO@Ag@PPy hybrid composites possess better cycle performance than bare ZnO at high charge/discharge rate.

**Figure 7:**
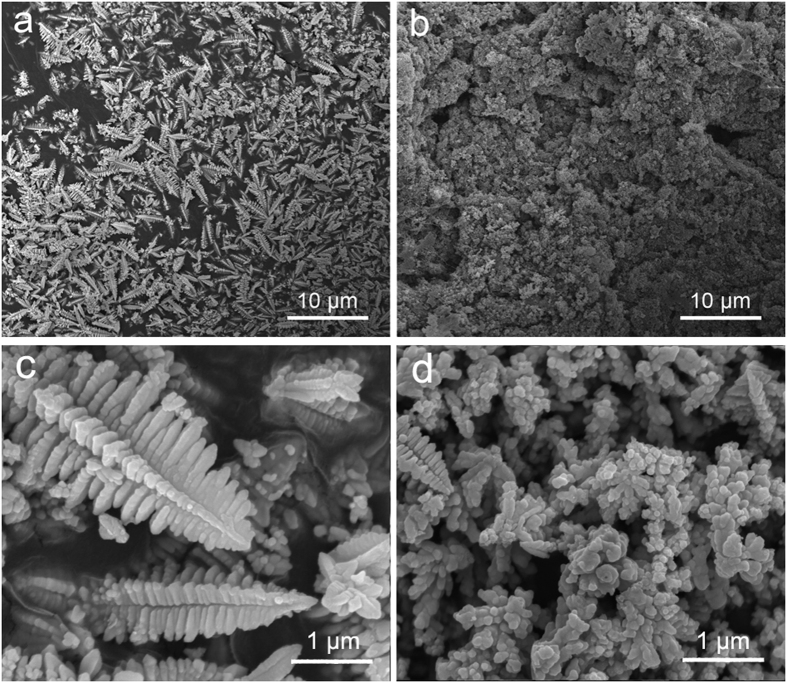
SEM images of (**a**) bare ZnO and (**b**) Sample 2 after 100 cycles at 1C rate, SEM images of (**c**) bare ZnO and (**d**) Sample 2 after 100 cycles at 3C rate.

Figure 7a,b show the SEM morphology of bare ZnO and sample 2 after 100 cycles (at 1C rate) respectively. Serious zinc dendrite can be observed while sample 2 exhibits grain morphology which is beneficial to the uniform distribution of current density. So it can be apparently concluded that the hybrid composites inhibit the dendrite formation effectively. Figure 7c,d show the morphology of bare ZnO and sample 2 after 100 cycles (at 3C rate) respectively. From Fig. 7c we know that the dendrite was well formed for bare ZnO, but there is little dendrite in Fig. 7d, demonstrating the superiority of the hybrid composite.

### Electrochemical impedance spectroscopy analysis

**Figure 8:**
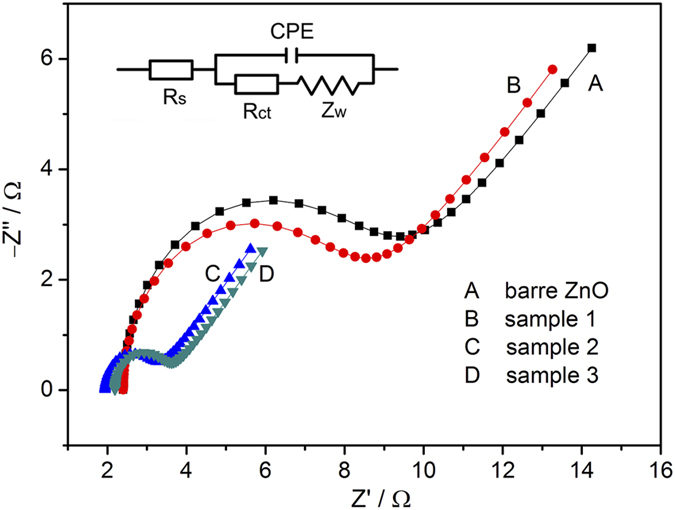
Electrochemical impedance spectroscopy and Randles equivalent circuit for bare ZnO (curve A), Sample 1 (curve B), Sample 2 (curve C) and Sample 3 (curve D).

In order to investigate the electrode reaction kinetics, electrochemical impedance spectroscopy analysis was conducted at 100% state-of-charging. The corresponding Nyquist plots of bare ZnO and hybrid composite electrodes are shown in Fig. 8. The Nyquist plot consists of a semicircle in the higher frequency and a straight tail in the lower frequency. Generally, the arc radius of semicircle reflects the magnitude of charge-transfer resistance (*R*_ct_), and the straight tail represents the Warburg impedance (*Z*_w_) of electrode, which is caused by the semi-infinite diffusion. Here, Randles–Ershler type equivalent circuit has been employed to fit the Nyquist plots, and the composition of the equivalent circuit can be found in the insert of Fig. 8. On the basis of the equivalent circuit, the value of *R*_ct_ can be calculated, which is 6.1 Ω, 5.3 Ω, 1.2 Ω and 1.2 Ω for bare ZnO electrode, sample 1, sample 2 and sample 3 electrode, respectively. The lower *R*_ct_ implies that the electron transfer is improved, and the kinetics process of electrochemical reaction is facilitated. In other hand, the Warburg impedance for bare ZnO, sample 1, sample 2 and sample 3 electrode is 0.04 Ω, 0.05 Ω, 0.07 Ω and 0.08 Ω respectively, indicating that the decoration on the surface of ZnO particle exerts some influence on the diffusion resistance between the electrolyte and electrode. But in summary, the disadvantage from decoration is overwhelmed by the advantage caused by decoration.

## Conclusions

ZnO@Ag@PPy hybrid composite is successfully prepared through a facile one-pot approach, in which silver-ammonia solution serves as oxidizing agent, and pyrrole monomer acts as reducing agent. FT-IR spectra and XRD pattern demonstrate that pyrrole has been successfully oxidized to polypyrrole while silver-ammonia complex ion has been reduced to metallic silver. The SEM and TEM images of hybrid composite reveal the composition and structure of composite, in which the surface of bare ZnO particles are successfully decorated with small metallic silver grains coated with polypyrrole layer. As-prepared hybrid composites provide better cycle performance than bare ZnO when estimated as anode material in zinc-nickel rechargeable battery. The improvements can be ascribed to follow reasons: (1) In the charge process, the insoluble metallic silver and polypyrrole improve the distribution of current density, which leads to uniform deposition of zincate ions, finally suppressing the growth of zinc dendrite. (2) The good conductivity of metallic silver also enhances reversibility of hybrid composite. In addition, the re-complexation of polypyrole with zincate ions could be another factor for the improvement of electrochemical performance.

## Additional Information

**How to cite this article**: Huang, J. *et al.* A novel ZnO@Ag@Polypyrrole hybrid composite evaluated as anode material for zinc-based secondary cell. *Sci. Rep.* **6**, 24471; doi: 10.1038/srep24471 (2016).
